# The EnvZ/OmpR Two-Component System Regulates the Antimicrobial Activity of TAT-RasGAP_317-326_ and the Collateral Sensitivity to Other Antibacterial Agents

**DOI:** 10.1128/spectrum.02009-21

**Published:** 2022-05-17

**Authors:** Maria Georgieva, Tytti Heinonen, Simone Hargraves, Trestan Pillonel, Christian Widmann, Nicolas Jacquier

**Affiliations:** a Institute of Microbiology, University Hospital Center and University of Lausanne, Lausanne, Switzerland; b Department of Biomedical Sciences, University of Lausanne, Lausanne, Switzerland; Emory University School of Medicine

**Keywords:** antimicrobial peptide, antibiotic resistance, two-component system, *Escherichia coli*, TAT-RasGAP_317-326_

## Abstract

The rapid emergence of antibiotic-resistant bacteria poses a serious threat to public health worldwide. Antimicrobial peptides (AMPs) are promising antibiotic alternatives; however, little is known about bacterial mechanisms of AMP resistance and the interplay between AMP resistance and the bacterial response to other antimicrobials. In this study, we identified Escherichia coli mutants resistant to the TAT-RasGAP_317-326_ antimicrobial peptide and found that resistant bacteria show collateral sensitivity to other AMPs and antibacterial agents. We determined that resistance to TAT-RasGAP_317-326_ peptide arises through mutations in the histidine kinase EnvZ, a member of the EnvZ/OmpR two-component system responsible for osmoregulation in E. coli. In particular, we found that TAT-RasGAP_317-326_ binding and entry is compromised in E. coli peptide-resistant mutants. We showed that peptide resistance is associated with transcriptional regulation of a number of pathways and EnvZ-mediated resistance is dependent on the OmpR response regulator but is independent of the OmpC and OmpF outer membrane porins. Our findings provide insight into the bacterial mechanisms of TAT-RasGAP_317-326_ resistance and demonstrate that resistance to this AMP is associated with collateral sensitivity to other antibacterial agents.

**IMPORTANCE** Antimicrobial peptides (AMP) are promising alternatives to classical antibiotics in the fight against antibiotic resistance. Resistance toward antimicrobial peptides can occur, but little is known about the mechanisms driving this phenomenon. Moreover, there is limited knowledge on how AMP resistance relates to the bacterial response to other antimicrobial agents. Here, we address these questions in the context of the antimicrobial peptide TAT-RasGAP_317-326_. We show that resistant Escherichia coli strains can be selected and do not show resistance to other antimicrobial agents. Resistance is caused by a mutation in a regulatory pathway, which lowers binding and entry of the peptide in E. coli. Our results highlight a mechanism of resistance that is specific to TAT-RasGAP_317-326_. Further research is required to characterize these mechanisms and to evaluate the potential of antimicrobial combinations to curb the development of antimicrobial resistance.

## INTRODUCTION

Currently, antibiotics are our first line of defense against bacterial pathogens but due to increasing rates of bacterial resistance, we are running out of effective antibiotics. The development of alternative antimicrobial treatments can help address this growing public health issue. Antimicrobial peptides (AMPs) are one such alternative that can supplement already existing antibiotic therapies or that can completely replace antibiotics (in cases where antibiotic resistance is a concern).

AMPs are short peptides with antibacterial activity (1). They were first described as naturally occurring peptides found in many prokaryotic and eukaryotic organisms where they participate in the innate defense against microbial pathogens (2). Because AMPs are quite diverse and have multiple distinct targets, they are an ideal source to further develop novel antibacterial agents. Interest in AMPs is underscored by reports of many being tested in clinical trials (3). However, despite the clear clinical potential of AMPs, we need to contemplate the fact that we still have limited understanding regarding two outstanding questions: (i) what are the mechanisms of bacterial resistance to AMPs (4, 5), and (ii) what is the association between peptide resistance and susceptibility to other antibacterial agents (2, 6–8). In the present work, we addressed these questions using the TAT-RasGAP_317-326_ AMP (9). This compound consists of the HIV TAT_48-57_ cell-penetrating peptide fused to a 10 amino acid sequenced derived from the Src homology 3 domain of human p120 RasGAP (10). TAT-RasGAP_317-326_ was initially described as an anti-cancer molecule (10–16). It was then found to also bear antimicrobial and antibiofilm activities (9, 17).

In a recent study, we reported specific resistance toward this peptide in several bacterial species, without collateral resistance to other AMPs (18). We thus decided to investigate the causes of such resistance using Escherichia coli as a model organism. First, we performed laboratory evolution of E. coli subjected to increasing concentrations of TAT-RasGAP_317-326_. We obtained strains that were resistant to this peptide but not to other AMPs or antibiotics. Using whole genome sequencing and bacterial genetics, we showed that mutations in the *envZ* gene, which codes for the inner-membrane sensor of the EnvZ/OmpR two-component system, are responsible for resistance against TAT-RasGAP_317-326_. Finally, we demonstrated that this resistance was specifically mediated by the OmpR regulator and was associated with reduced peptide binding and entry in bacterial cells.

## RESULTS

### Laboratory-evolved TAT-RasGAP_317-326_-resistant E. coli strains show collateral sensitivity to other AMPs and antibiotics.

We previously found that bacteria exposed to gradually increasing concentrations of the antimicrobial TAT-RasGAP_317-326_ peptide develop resistance toward this peptide with a MIC increase of several fold compared to the wild-type parental strains (18). To understand the mechanisms underlying this resistance phenomenon, we focused on a representative set of four E. coli clones (named mutants A to D) that were isolated after the E. coli MG1655 parental strain was exposed to increasing concentrations of TAT-RasGAP_317-326_ over 12 passages (Table 1).

**TABLE 1:** Description of four individual TAT-RasGAP_317-326_-resistant strains obtained by selection with increasing concentrations of peptide^*a*^

Mutations detalis/antimicrobial agents	MIC/IC_50_	MG1655	Mutant A	Mutant B		Mutant C		Mutant D	
Mutated gene(s)			*envZ*	*envZ*	*basS*	*envZ*	*rfaY*	*envZ*	*yrbL*
Locus tag			b3404	b3404	b4112	b3404	b3625	b3404	b3207
Mutated nucleotides			G697A	T722G	A371C	T819G	649delA	C742G	G445C
Mutated amino acids			D233N	V241G	E124A	D273E	L226Stop	P248A	V149L
TAT-Ras GAP_317-326_	MIC (μM)	4/8	24/24	24/24		24/24		48/48	
	IC_50_ (μM)	2.11/5.35	16.7/21	13.3/19.5		19.0/19.4		23.8/29.3	
Polymyxin B	MIC (μg/mL)	1.5/2	1/2	1/2		1.5/2		1/2	
	IC_50_ (μg/mL)	0.43/1.10	0.41/0.73	0.66/1.25		0.94/0.95		0.54/0.93	
Melittin	MIC (μg/mL)	32/32	20/24	24/32		32/32		24/32	
	IC_50_ (μg/mL)	25/25.2	16.5/20.4	20.1/24.1		24.2/25.9		20.5/23.6	
SDS	MIC (μg/mL)	512/512	512/1024	512/1024		512/512		512/512	
	IC_50_ (μg/mL)	166/169	75/80	129/144		141/158		148/170	
Gentamicin	MIC (μg/mL)	12/16	6	4/6		6/8		2/3	
	IC_50_ (μg/mL)	2.67/5.72	2.64/2.66	2.61/2.67		2.67/3.34		0.92/1.97	
Aztreonam	MIC (ng/mL)	62.5/125	125/250	125/125		125/250		125/125	
	IC_50_ (ng/mL)	26/38	60/70	47/48		39/42		32/37	
Tetracycline	MIC (μg/mL)	2/4	2/4	2/4		2/4		2/4	
	IC_50_ (μg/mL)	0.33/0.37	0.48/0.51	0.40/0.43		0.40/0.40		0.37/0.39	
Meropenem	MIC (ng/mL)	31.25/31.25	125/62.5	31.25/62.5		31.25/62.5		62.5/62.5	
	IC_50_ (ng/mL)	10.7/13.5	27.6/17.4	17.3/20.9		17.8/23.6		16.4/19.3	

a Mutations detected in each strain by comparison with the parental MG1655 E. coli strain are listed. MIC and IC_50_ of TAT-RasGAP_317-326_, polymyxin B, melittin, SDS, gentamicin, aztreonam, tetracycline, and meropenem against each strain are listed. The results of two independent experiments are shown.

**FIG 1:**
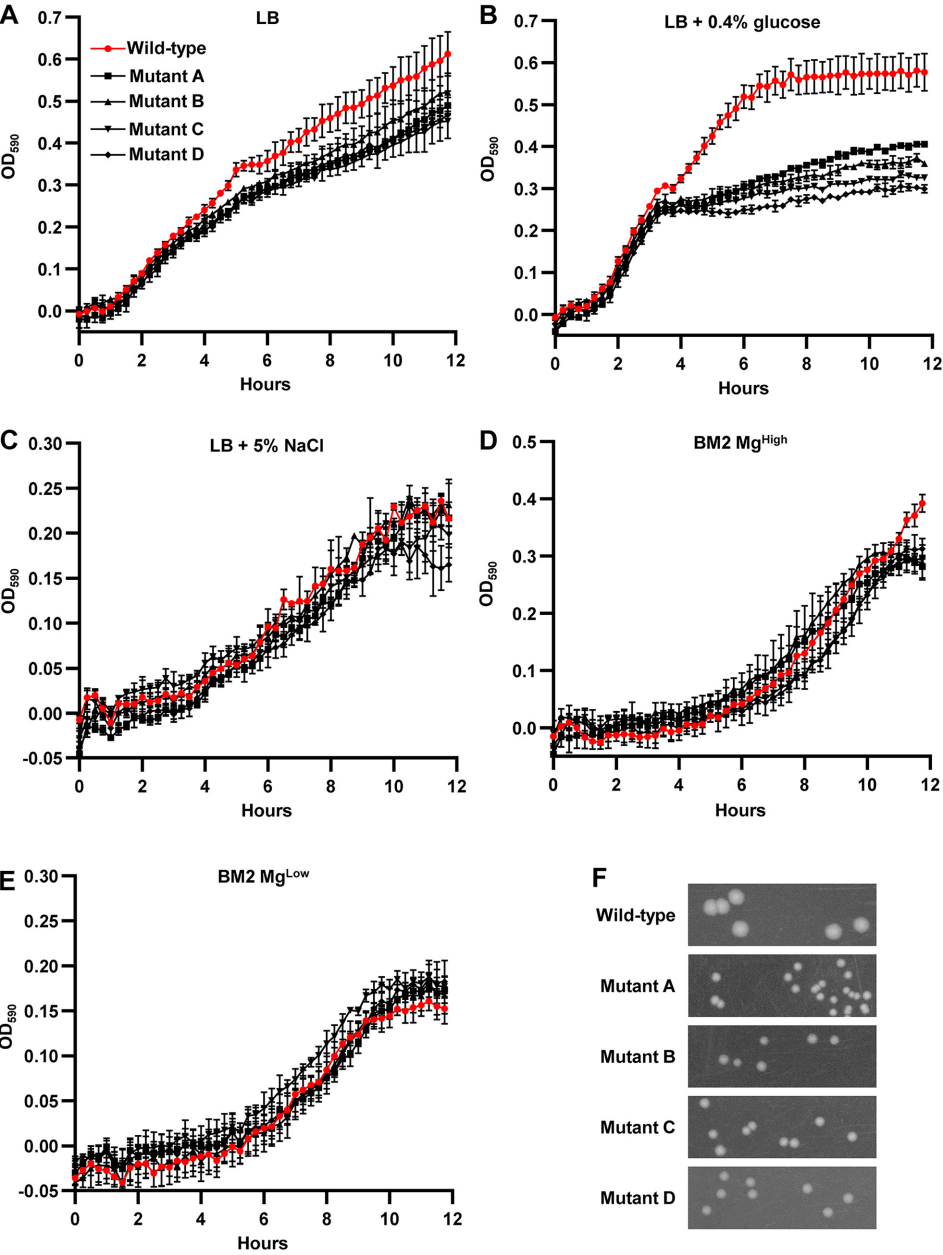
Growth curve analysis of E. coli mutants resistant to TAT-RasGAP_317-326_ peptide. (A to E) Resistant strains show growth defect in rich medium supplemented with glucose, but not in the other media tested. Growth of the indicated strains was monitored by spectrophotometry at the indicated time points. Cultures were grown overnight and then diluted to OD_600_ = 0.01 in the indicated medium at time zero. Error bars show standard deviation of three independent experiments. (F) Colonies of resistant mutants on LB agar plates were smaller than wild-type colonies. Indicated strains were grown overnight in LB at 37°C and then diluted to OD_600_ = 0.1. Each bacterial culture was spotted on LB agar plates and incubated overnight at 37°C 6 h after dilution.

First, we characterized and compared the growth rate of these mutants to that of the wild-type strain. Growth rates during exponential phase were comparable between wild-type and all four mutant strains in Luria-Bertani (LB) broth (Fig. 1A). However, all four mutants differed phenotypically from the wild-type strain as they formed smaller colonies on LB agar (Fig. 1F). Also, in the presence of 0.4% glucose in LB broth, all four mutants exhibited a growth defect (Fig. 1B). In contrast, no growth defect was observed in rich medium supplemented with 5% NaCl or in defined media such as BM2 with high (2 mM) or low (20 μM) concentrations of magnesium sulfate (Fig. 1C to E).

**FIG 2:**
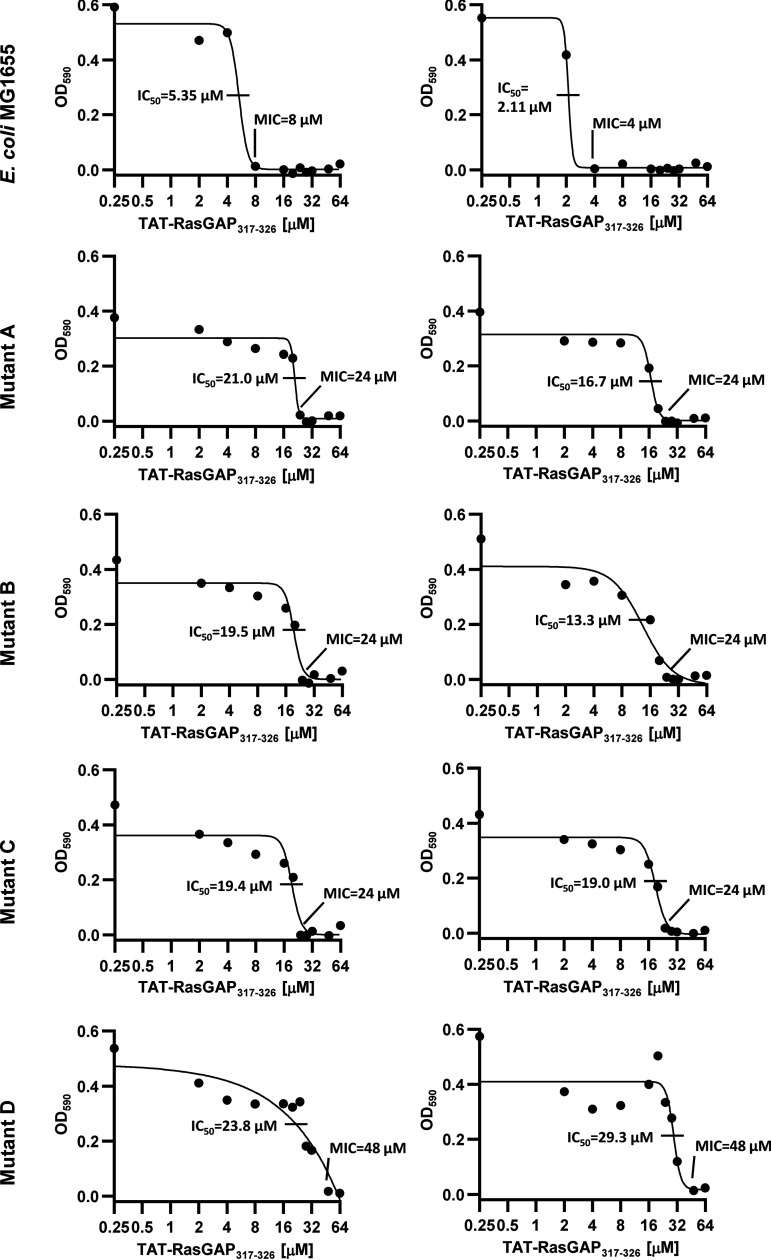
Laboratory-evolved E. coli mutants are resistant to TAT-RasGAP_317-326_ peptide. Indicated strains were grown overnight, diluted to OD_600_ = 0.1 and grown for 1 h. Culture was then diluted 200 times in sequential dilutions of TAT-RasGAP_317-326_. Growth was measured by spectrophotometry after overnight incubation at 37°C. MIC was determined as the lowest concentration resulting in an undetectable growth. IC_50_ was calculated using GraphPad software. Two independent experiments are shown for each strain.

Next, we analyzed the characteristics of these four mutants with regard to their resistance against TAT-RasGAP_317-326_ and other antibacterial agents. The MIC of TAT-RasGAP_317-326_ was higher in the four mutants (24-48 μM) compared to the corresponding MIC of the wild-type E. coli strain (4 to 8 μM, Table 1; Fig. 2). To study whether TAT-RasGAP_317-326_ resistance leads to cross-resistance or collateral sensitivity toward other antibacterial agents, we measured the susceptibility of the four mutants to other peptides and antibiotics. We found no significant difference between the wild-type E. coli strain and mutants A-D with regard to the MIC of the polymyxin B and melittin AMPs, the sodium dodecyl sulfate (SDS) detergent, and the tetracycline, aztreonam, and meropenem antibiotics (Table 1). This indicates that cross-resistance between TAT-RasGAP_317-326_ and these antibacterial compounds is limited. Conversely, the MIC of gentamicin was decreased for all four mutants compared to the wild-type strain, showing that acquisition of resistance toward TAT-RasGAP_317-326_ can induce collateral sensitivity to some antimicrobial agents.

### Mutations in the EnvZ sensor confer resistance to TAT-RasGAP_317-326_.

We next set out to identify the mutations underlying resistance toward TAT-RasGAP_317-326_. To do this, we performed whole genome sequencing of mutants A to D and compared these sequences with that of the unevolved parental E. coli. Single nucleotide polymorphisms (SNPs) found in the four independent mutants are listed in Table 1. All resistant strains possessed non-synonymous mutations in *envZ*, a gene coding for a histidine kinase functioning as the inner membrane sensor of the EnvZ/OmpR two-component system. This two-component system is involved in osmoregulation in E. coli where EnvZ controls the activity of the OmpR transcription factor, which in turn controls the expression of several downstream genes (19). All mutations we found (D233N, V241G, D273E, and P248A) are located within the cytoplasmic dimerization domain of EnvZ, which is involved in protein dimerization and phospho-group transfer from EnvZ to the OmpR response regulator protein (20). Three of the strains also contained an additional mutation in other genes (*basS*, *rfaY*, and *yrbL*). The gene *basS* codes for the histidine kinase sensor of the BasRS two-component system (21), while *yrbL* codes for a protein of unknown function transcriptionally regulated by variations of Mg^2+^ levels (22). We previously discussed the potential role of *rfaY*, coding for a protein involved in lipopolysaccharide (LPS) biosynthesis, in the bacterial response to TAT-RasGAP_317-326_ (18). Due to the prevalence of the *envZ* gene mutations in mutants A to D, we decided to investigate the role of EnvZ in bacterial resistance toward TAT-RasGAP_317-326_. We selected mutant A (hereby referred to as EnvZ^D233N^) for further investigation, because this strain contained no other mutations.

### Transcriptomic analysis highlights gene expression modulation in E. coli EnvZ^D233N^.

**FIG 3:**
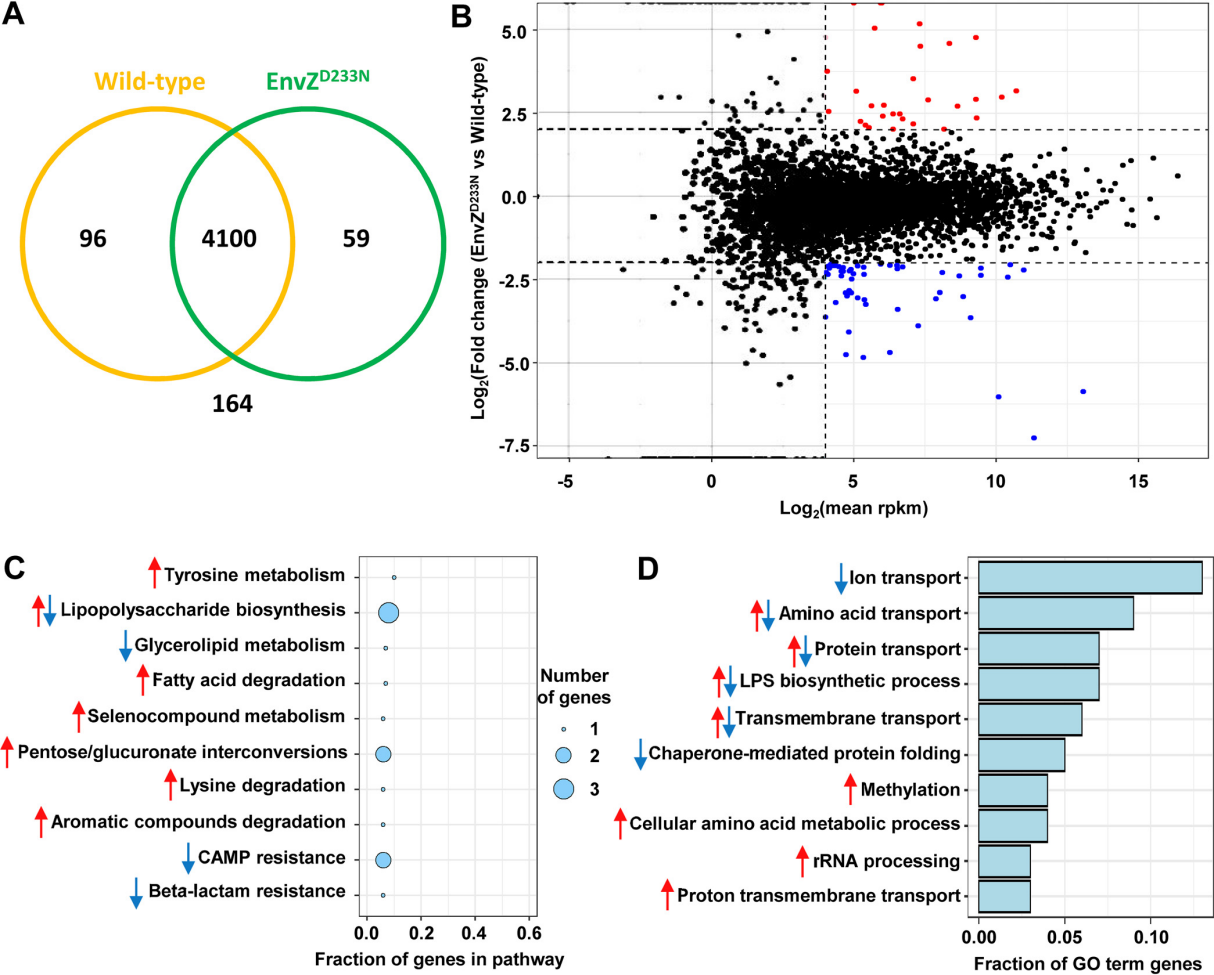
Effect of the EnvZ^D233N^ mutation on the E. coli transcriptome. (A) A large majority of predicted genes were detected in both wild-type and EnvZ^D233N^ strains by RNA-sequencing. Number of genes, which expression was detected in wild-type strain, in EnvZ^D233N^ strain, in both or in none is shown. (B) Comparison of gene expression between wild-type and EnvZ^D233N^ strains. Genes detected by RNA-Sequencing were classified regarding mean of their abundance. This was expressed as the Log_2_ of reads per kilobase of transcript per million mapped reads (Log_2_RPKM), in order to take into account the length of the different transcripts. Abundance of mRNAs was compared between the EnvZ^D233N^ mutant and the wild-type and expressed by MA plot as the Log_2_ of the fold change along the *y* axis and normalized gene expression along the *x* axis. Genes upregulated in the mutant versus wild-type strain are highlighted in red (log_2_ fold change > 2). Genes downregulated in the mutant versus wild-type strain are highlighted in blue (log_2_ fold change < −2). Genes with a low average expression (an arbitrary threshold was set at log_2_(rpkm) = 4) were not considered in subsequent analyses. (C) Selection of the 10 KEGG pathways containing the highest proportion of genes, whose expression is modulated in EnvZ^D233N^ mutant relative to the wild-type strain. (D) Classification of GO term classes (biological processes) which contain the highest fraction of modulated genes. In (C) and (D), arrows indicate whether the category contains genes either upregulated (red) or downregulated (blue) in the mutant relative to wild type.

The EnvZ/OmpR two-component regulatory system regulates the expression of a large set of genes (23). Differential gene expression might explain, to some extent, the resistance phenotype conferred by the EnvZ mutant. To explore which genes might be involved in peptide resistance of the EnvZ mutant, we therefore compared the transcriptome profiles of wild-type and EnvZ^D233N^ E. coli (Data set 1). In total, we detected expression of 4,255 genes, representing 96.3% of the genes found in E. coli. Among them, 4,100 were shared between the wild-type and EnvZ^D233N^ strains (Fig. 3A). First, we set a threshold to exclude genes with low expression, shown as a vertical dashed line in Fig. 3B. Then, we classified upregulated genes as those being expressed more than 4-fold more in the EnvZ^D233N^ mutant than in the wild-type strain (red dots in Fig. 3B; Table S2). Similarly, genes being expressed at least four times less in the EnvZ^D233N^ mutant than in the wild-type strain were classified as downregulated (blue dots in Fig. 3B; Table S3). Altogether, 81 genes were differently expressed in the EnvZ^D233N^ mutant. KEGG pathway and Gene Ontology (GO) term analyses of these genes showed that they predominantly belong to metabolic pathways, such as LPS biosynthesis and transport of ions, amino acids, and proteins (Fig. 3C and D). In Fig. 3C and D, arrows indicate whether the category contains genes that are either upregulated or downregulated in the mutant relative to the wild-type strain.

**FIG 4:**
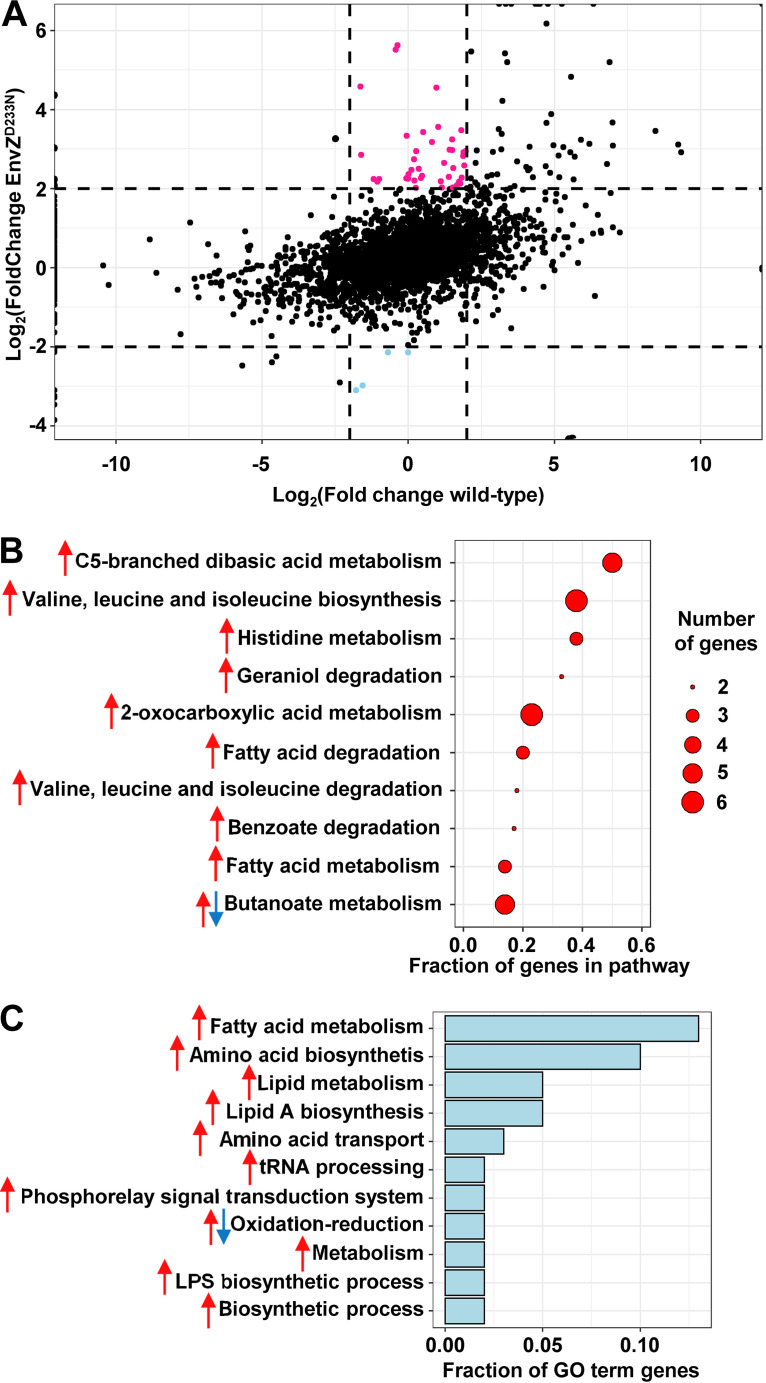
Transcriptional response to TAT-RasGAP_317-326_ treatment differs between wild-type and EnvZ^D233N^ strain. RNA of exponentially growing bacteria (wild-type and EnvZ^D233N^ mutant) was extracted and RNA-sequencing was performed with, and without, a 1-h treatment with 10 μM TAT-RasGAP_317-326_. (A) Genes were classified regarding their relative abundance in conditions tested. Abundance of mRNAs was compared between the EnvZ^D233N^ mutant treated or not with 10 μM TAT-RasGAP_317-326_ and the wild-type in same conditions and expressed as the Log_2_ of the fold change (treated versus untreated). Genes for which Log_2_ fold change is higher than 2 for EnvZ^D233N^, but not for wild-type, are highlighted in pink and are considered as specifically upregulated in the mutant upon peptide treatment. Genes with Log_2_ fold change lower than −2 in the EnvZ^D233N^ strain, but not in the wild-type are highlighted in blue and considered as genes specifically downregulated in the mutant upon peptide treatment. (B) Selection of the 10 KEGG pathways containing the highest proportion of genes, whose expression is modulated in peptide-treated EnvZ^D233N^ mutant but not in the wild-type treated with peptide. (C) Classification of GO term classes (biological processes) which contain the highest fraction of genes, whose expression is modulated in peptide-treated EnvZ^D233N^ mutant but not in the wild-type treated with peptide. In (B) and (C), arrows indicate whether the category contains genes either upregulated (red) or downregulated (blue) in the mutant relative to wild-type upon treatment with the peptide.

We also performed RNA sequencing to study the effect of TAT-RasGAP_317-326_ on the E. coli transcriptome (Fig. 4; Data set 1). We compared the log fold change of the two groups, wild-type bacteria with peptide and EnvZ^D233N^ mutant with peptide, each against a control, wild-type bacteria in the absence of peptide and EnvZ^D233N^ mutant in the absence of peptide, respectively (Fig. 4A). We highlighted genes whose expression was increased in the presence of the peptide in the resistant mutant but that showed no change in the wild-type (Fig. 4A, pink dots; Table S4). We also highlighted genes whose expression was decreased after exposure of the EnvZ^D233N^ mutant to the peptide and which showed no change in the wild-type bacteria (Fig. 4A, blue dots; Table S5). All genes showing differential expression between the wild-type strain and the EnvZ^D233N^ mutant upon TAT-RasGAP_317-326_ treatment were grouped according to KEGG pathway and GO term analyses (Fig. 4B and C). We found that the modulated pathways belonged to metabolic processes such as fatty acid degradation, amino acid biosynthesis, and LPS biosynthesis. Taken together, our results in Fig. 3 and 4 indicate that the EnvZ^D233N^ mutation itself causes a wide transcriptional modification compared with wild-type E. coli and that additional genes are modulated in this strain in the presence of TAT-RasGAP_317-326_ peptide.

### Whole gene deletion of *envZ* does not promote TAT-RasGAP_317-326_ resistance.

**FIG 5:**
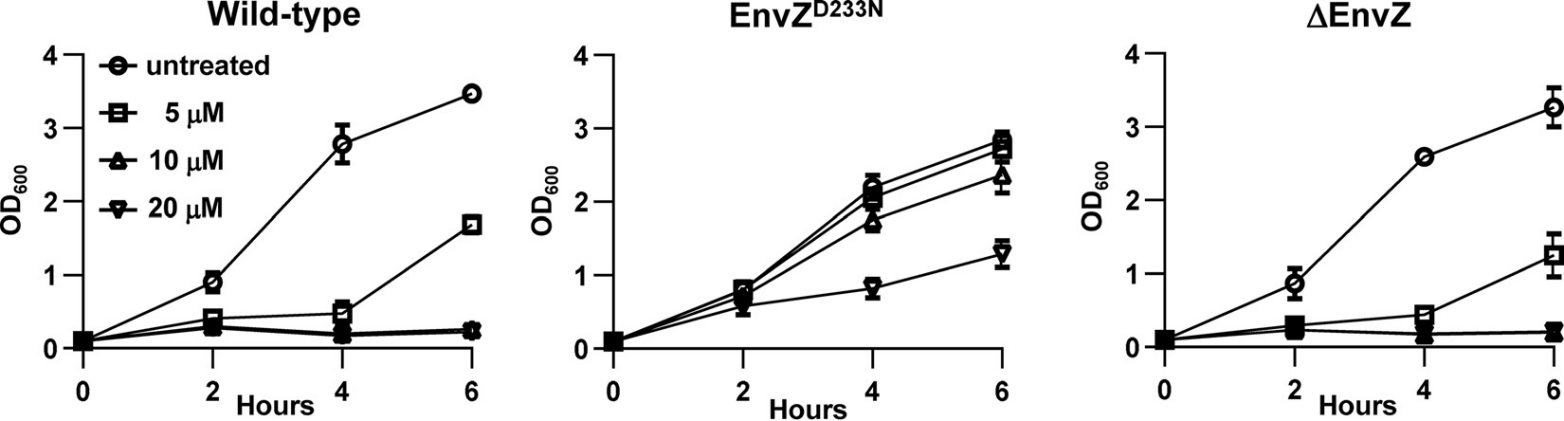
Whole gene deletion of EnvZ does not confer TAT-RasGAP_317-326_ resistance. E. coli strains wild-type, EnvZ^D233N^ or ΔEnvZ were grown overnight in LB at 37°C, diluted to OD_600_ = 0.1 in the same medium, and after 1 h of incubation at 37°C, the indicated concentrations of TAT-RasGAP_317-326_ were added. Growth was measured by spectrophotometry at the indicated time points. Error bars show ranges of two independent experiments.

Point mutations can have a variety of effects on protein function. To determine whether the single amino acid mutation D233N in the EnvZ protein is sufficiently disruptive as to correspond to a null *EnvZ* mutant, we compared the phenotypes of the EnvZ^D233N^ and EnvZ deletion (ΔEnvZ) strains in the presence of TAT-RasGAP_317-326_ (Fig. 5). The effect of TAT-RasGAP_317-326_ against the ΔEnvZ strain was comparable with that against wild-type E. coli, indicating that while the EnvZ^D233N^ amino acid mutation promotes peptide resistance, the complete removal of EnvZ activity (via whole gene deletion) does not. Thus, our data indicate that the D233N substitution is a gain-of-function mutation conferring resistance to TAT-RasGAP_317-326_.

### The EnvZ^D233N^ single amino acid mutation autonomously confers resistance to TAT-RasGAP_317-326_.

**FIG 6:**
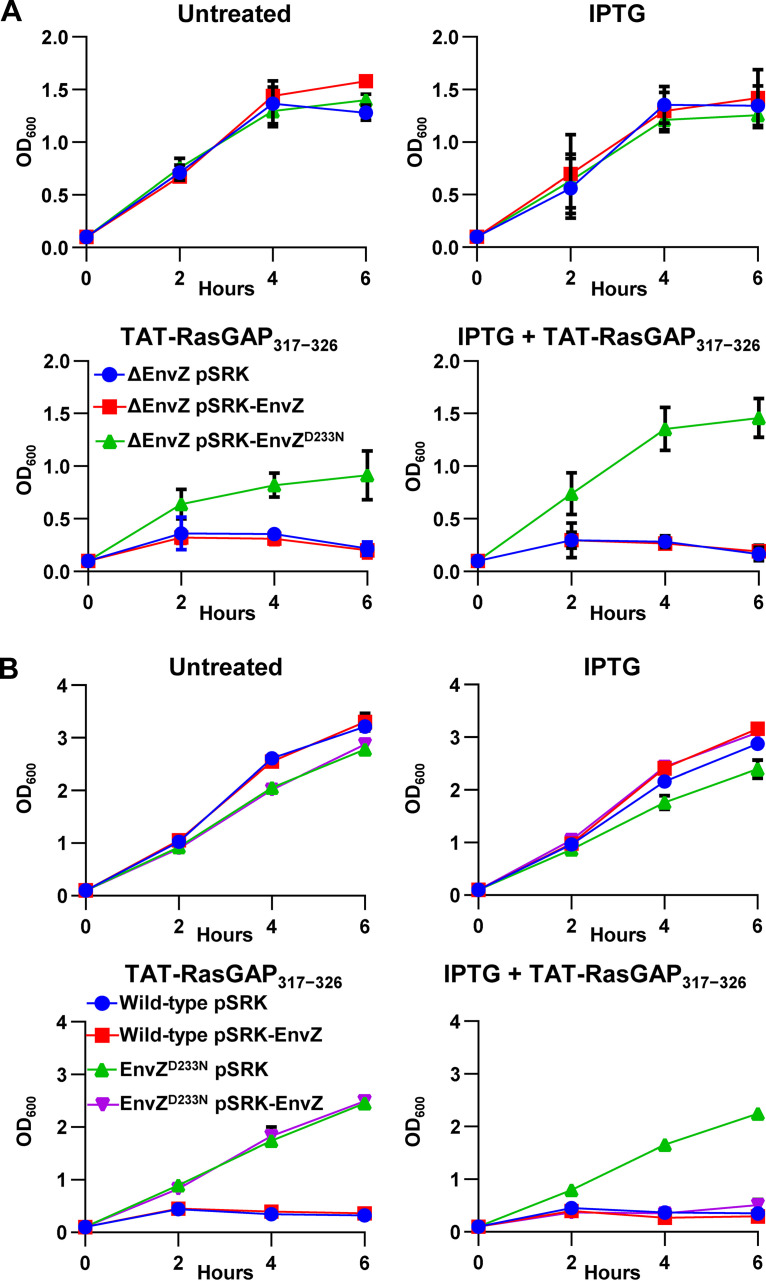
EnvZ^D233N^ point mutation is sufficient for resistance to TAT-RasGAP_317-326_ peptide, but sensitivity can be restored by overexpression of the wild-type version of EnvZ. (A) Growth curve analysis comparing three separate ΔEnvZ E. coli strains with IPTG-inducible plasmid pSRK, pSRK containing wild-type *envZ* gene (pSRK-EnvZ), or point mutant *envZ* gene (pSRK-EnvZ^D233N^). Panels show ΔEnvZ E. coli strains grown in the presence or in the absence of 5 μM TAT-RasGAP_317-326_ with or without 1 mM IPTG, as indicated. Strains were grown overnight in LB, diluted to OD_600_ = 0.1 in the same medium, and incubated 1 h before addition of 10 μM TAT-RasGAP_317-326_. Growth was monitored by spectrophotometry at the indicated time points. (B) Wild-type or EnvZ^D233N^ mutant with IPTG-inducible plasmid pSRK or pSRK containing wild-type *envZ* gene (pSRK-EnvZ) were diluted to 0.1 OD_600_ and grown in the presence or in the absence of 10 μM TAT-RasGAP_317-326_ with or without 1 mM IPTG, as indicated. Growth was monitored by OD_600_ measurements at the indicated time points. Average and range of two independent experiments are shown.

To gain further insight into the role of the EnvZ^D233N^ mutation for peptide resistance, we overexpressed this mutation from an IPTG-inducible promoter in an E. coli *envZ* deletion strain (ΔEnvZ) (24). We found that overexpression of EnvZ^D233N^ but not wild-type EnvZ promoted E. coli growth in the presence of 10 μM TAT-RasGAP_317-326_ (Fig. 6A). Our findings indicate that the EnvZ^D233N^ single amino acid mutation is sufficient to promote E. coli resistance to the antimicrobial TAT-RasGAP_317-326_ peptide. While Fig. 6A clearly demonstrates the autonomous ability of the EnvZ^D233N^ mutation to confer resistance to TAT-RasGAP_317-326_, we found that overexpressing the wild-type EnvZ protein in the EnvZ^D233N^ strain restored sensitivity of the bacteria to the peptide (Fig. 6B).

### TAT-RasGAP_317-326_ binding and entry are compromised in E. coli EnvZ^D233N^ mutant.

**FIG 7:**
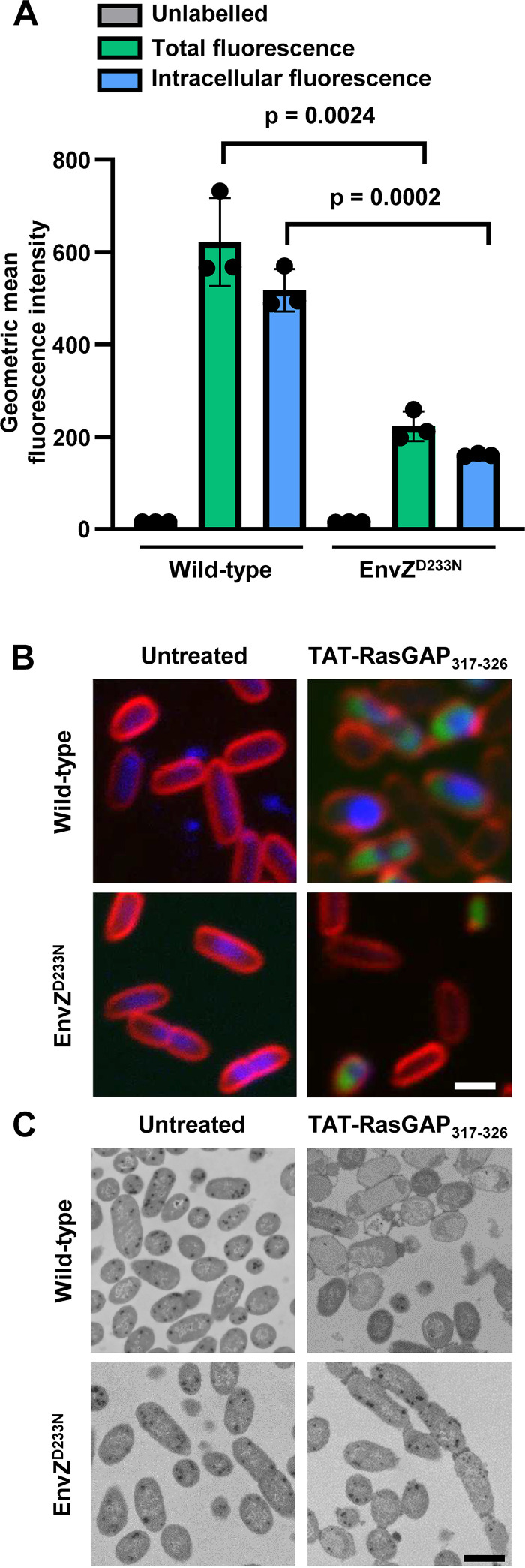
Peptide binding and entry is reduced in EnvZ^D233N^ mutant in comparison to wild-type E. coli. (A) Flow cytometry to measure peptide binding and entry to bacterial cells. Geometric mean of fluorescence intensity of each strain after exposure to 10 μM FITC-labeled TAT-RasGAP_317-326_ peptide is shown (green panel, total fluorescence). Trypan blue was added to quench extracellular fluorescence and quantify peptide entry (blue panel, intracellular fluorescence). *P* values shown are obtained from *t* test statistical analysis. (B) Fluorescence microscopy of bacterial cells (wild-type and EnvZ^D233N^ E. coli) treated or not with 10 μM FITC-labeled TAT-RasGAP_317-326_ peptide indicates that fluorescent peptide accumulates less in the EnvZ^D233N^ mutant than in the wild type. Bacterial membranes were labeled with 5 μg/mL FM4-64 (red) and fixed with 4% paraformaldehyde solution before staining of DNA with DAPI (blue). Samples were imaged using a Zeiss LSM710 confocal microscope and analyzed using ImageJ software. Bar = 2 μm. (C) Electron microscopy of wild-type and EnvZ^D233N^ E. coli highlights the differences of morphologies between these two strains upon treatment with 10 μM TAT-RasGAP_317-326_. Bacteria treated with the peptide for 1 h were then fixed using glutaraldehyde and prepared for electron microscopy. Samples were imaged via transmission electron microscopy. Images were treated using ImageJ software. Bar = 2 μm.

We measured binding and entry of TAT-RasGAP_317-326_ peptide into wild-type bacteria and EnvZ^D233N^ mutant by quantitating fluorescence of bacteria via flow cytometry after incubation with a FITC-labeled version of the peptide. In order to differentiate peptide surface binding to bacterial cells and peptide entry into bacteria, we first measured total fluorescent signal (i.e., the sum of surface and internalized peptide) (green panels in Fig. 7A). Subsequently, trypan blue was added to the same samples to quench the extracellular fluorescent signal and measure signal from intracellular fluorescence only (blue panels in Fig. 7A). Our data showed that peptide binding and entry is decreased in the EnvZ^D233N^ mutant in comparison to the wild-type strain (Fig. 7A). We also detected decreased peptide accumulation in the EnvZ^D233N^ mutant as assessed by fluorescence microscopy (Fig. 7B). We further compared the morphology of wild-type and mutant bacterial cells after peptide exposure via electron microscopy (Fig. 7C). We found that TAT-RasGAP_317-326_ induces morphological changes in wild-type E. coli while changes were less evident in the EnvZ^D233N^ mutant.

### Peptide resistance mediated by EnvZ^D233N^ is dependent on the response regulator OmpR but is independent of the outer membrane OmpC and OmpF porins.

**FIG 8:**
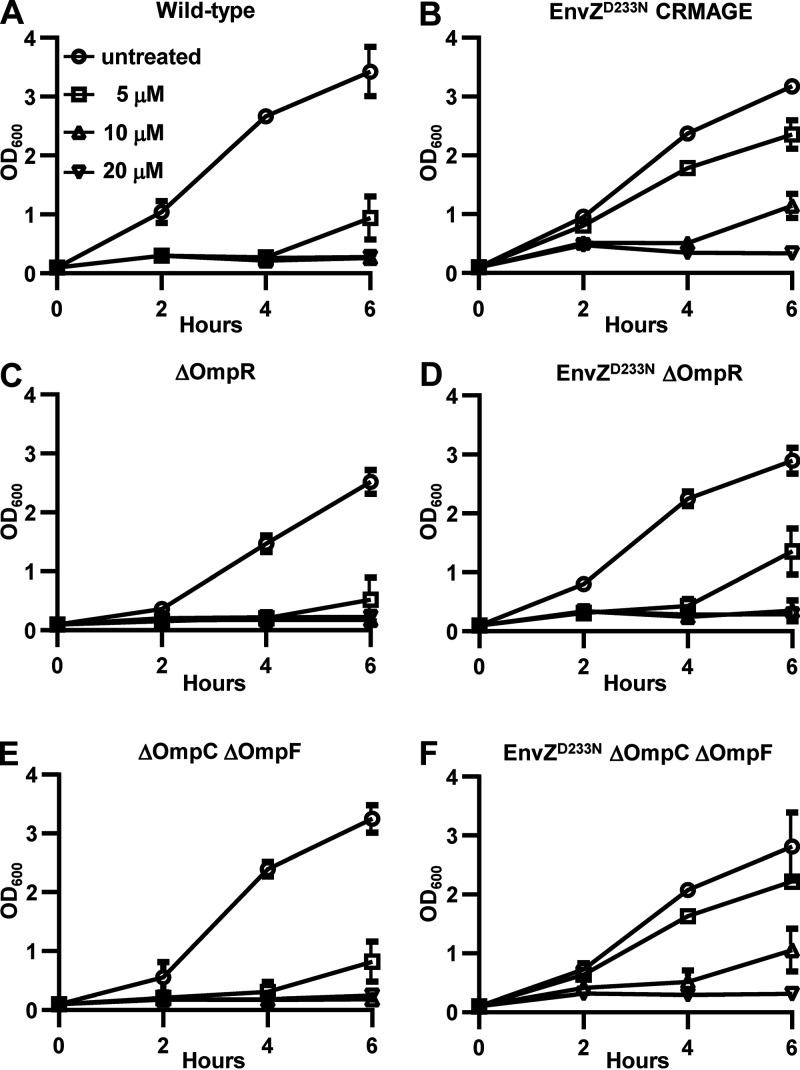
Peptide resistance is dependent on the response regulator OmpR. Growth curve analysis of indicated strains in the presence of TAT-RasGAP_317-326_ peptide. (A, B) Introduction of EnvZ^D233N^ mutation via Crispr-Cas9 technology in E. coli replicates the peptide-resistance phenotype of the laboratory-evolved mutants. (C, D) Deletion of the response regulator OmpR in EnvZ^D233N^ E. coli abolishes peptide resistance. (E, F) Deletion of OmpC and OmpF in EnvZ^D233N^ E. coli does not have an effect on peptide resistance. Indicated strains were grown overnight, diluted to OD_600_ = 0.1, grown for 1 h and treated or not with the indicated concentrations of TAT-RasGAP_317-326_. Data shown are average and range of two independent experiments.

EnvZ regulates the phosphorylation status of the transcriptional factor OmpR, which in turn modulates the expression of a variety of genes, among which are the outer membrane OmpC and OmpF porins (19). Porins mediate the passive diffusion of small chemicals across the outer membrane of Gram-negative bacteria. Because the porins OmpC/OmpF have been previously implicated in antibiotic resistance in E. coli (25), we wanted to evaluate their potential role in EnvZ^D233N^-mediated TAT-RasGAP_317-326_ resistance. We first introduced the *envZ^G697A^* mutation (the mutation that creates the EnvZ^D233N^ amino acid substitution) in the genome of E. coli using a CRISPR-Cas9 based approach (CRMAGE) (26). The presence of the desired mutation was validated by Sanger sequencing of the *envZ* gene. As expected, this strain was resistant to TAT-RasGAP_317-326_ (Fig. 8A and B). Next, we deleted *ompR* in both wild-type and EnvZ^D233N^ E. coli and compared the resistance profiles of these two strains. We found that E. coli EnvZ^D233N^ ΔOmpR is peptide-susceptible, suggesting that EnvZ^D233N^ functions through OmpR to promote peptide resistance (Fig. 8C and D). Finally, we created a strain harboring the EnvZ^D233N^ mutation in a background lacking both *ompC* and *ompF* genes (27). Absence of both OmpC and OmpF did not affect the resistance induced by the EnvZ^D233N^ mutation (Fig. 8E and F). Taken together, these results suggest that the EnvZ^D233N^ mutation functions through OmpR to promote TAT-RasGAP_317-326_ resistance, but does not require the OmpC and OmpF porins.

## DISCUSSION

AMPs are promising alternatives to antibiotics and many AMPs have reached advanced stages of testing in clinical trials (3). However, we still lack a good understanding of (i) how bacteria develop resistance toward AMPs and (ii) how AMP resistance impacts responses to other antibacterial agents. Here, we address these topics using TAT-RasGAP_317-326_, initially identified as an anti-cancer compound (10–16, 28, 29) and recently shown to be an effective antimicrobial peptide against a range of bacterial species (9, 17, 18).

First, we showed that we can derive E. coli strains resistant to TAT-RasGAP_317-326_ using laboratory evolution through continuous bacterial exposure to the peptide. Interestingly, we found that (i) bacterial resistance was restricted to TAT-RasGAP_317-326_, (ii) resistance to TAT-RasGAP_317-326_ led to gentamicin sensitivity, and (iii) there was no cross-resistance with the other AMPs or antibiotics tested here. The question of bacterial cross-resistance and cross-sensitivity is continually being investigated (30–39). Several studies, similar to ours, have identified a lack of cross-resistance and complex collateral sensitivity networks between AMPs and antibiotics. In contrast, other studies have highlighted the frequent emergence of bacterial cross-resistance which corroborates the complexity of these phenomena, the difficulty in making generalized conclusions, and the need to perform case-by-case investigations for each AMP of interest (8, 38, 40).

Through laboratory evolution, we isolated four E. coli TAT-RasGAP_317-326_ resistant mutants. We found that all four resistant clones have a growth defect in the presence of glucose, but not in other medium compositions. This data demonstrates that TAT-RasGAP_317-326_ resistance confers a variable fitness burden to mutants, and aligns with other studies demonstrating that resistance acquisition by bacteria is commonly associated with a fitness cost (8, 41).

Whole genome sequencing showed that TAT-RasGAP_317-326_-resistant E. coli strains have a point mutation in the sensor *envZ* histidine kinase, a member of the EnvZ/OmpR two-component regulatory system that is responsible for osmoregulation in E. coli. One hypothesis is that EnvZ mutations contribute to peptide resistance via alterations in the activity of EnvZ and the EnvZ/OmpR regulatory cascade. EnvZ is known to regulate the OmpR transcription factor via changes in its phosphorylation and dephosphorylation state. Indeed, two of the mutations that we detected in this study (V241G and P248A) are amino acids essential for the phosphatase activity of EnvZ (42). It is tempting to hypothesize that mutations such as these alter EnvZ/OmpR activity and expression of downstream target genes that in turn, regulate peptide resistance. Attractive candidates from the group of OmpR-dependent genes are OmpC and OmpF, which are controlled by OmpR and regulate bacterial outer membrane permeability (43, 44). Additionally, OmpC and OmpF have previously been implicated in antibiotic resistance in E. coli (25). However, deletions of *ompC* and *ompF* in the EnvZ^D233N^ background did not affect peptide resistance, suggesting that OmpC and OmpF are not involved in EnvZ-mediated TAT-RasGAP_317-326_ resistance. In contrast, OmpR is required for peptide resistance as a whole gene deletion of OmpR in EnvZ^D233N^ renders this E. coli strain peptide-susceptible.

Furthermore, we found that overexpression of the wild-type version of EnvZ from an IPTG-inducible promoter in EnvZ^D233N^ mutant background restored the sensitivity of this mutant to TAT-RasGAP_317-326_ (Fig. 6B). This data suggests that an excess of wild-type EnvZ protein, even in the presence of EnvZ^D233N^, restores E. coli susceptibility to the peptide. This is intriguing since we also show that the EnvZ^D233N^ mutation is sufficient to promote resistance to TAT-RasGAP_317-326_ in a ΔEnvZ E. coli deletion strain (Fig. 6A). It might be that EnvZ and EnvZ^D233N^ both compete for binding to the response regulator OmpR and mediate their effects via this interaction (consistent with our finding for OmpR dependence in the resistant phenotype). In circumstances where wild-type is in excess relative to the EnvZ^D233N^ mutant (such as in Fig. 6B), OmpR would be sequestered away from EnvZ^D233N^. Inversely, in circumstances where EnvZ wild-type is absent (Fig. 6A), EnvZ^D2333N^ would be able to bind OmpR and mediate peptide resistance.

As indicated above, the role of OmpR in EnvZ-mediated TAT-RasGAP_317-326_ resistance is clear but the OmpC and OmpF porins are not involved. Thus, it remains to be investigated which OmpR-regulated genes mediate this effect. Of particular interest are OmpR-dependent genes that belong to the LPS biosynthesis cluster. A recent study reported that AMP resistance in bacteria is controlled by the activity of WaaY, a kinase responsible for the phosphorylation of the inner core of LPS (35). Increased activity of WaaY leads to increased negative surface charge of the bacterial membrane and therefore increased susceptibility to antimicrobial peptides (which are in general positively charged) (35). A potential involvement of LPS-regulating genes in TAT-RasGAP_317-326_ resistance would be consistent with previous findings from our group that highlighted the importance of LPS integrity for the antibacterial activity of TAT-RasGAP_317-326_ (18). Similarly, here, we find significant expression changes in LPS-related genes in the EnvZ^D233N^ resistant mutant compared to wild-type E. coli both in the absence and presence of the peptide.

Mechanistically, peptide resistance is mediated via reduced binding and entry in the EnvZ^D233N^-resistant mutant compared with the parental wild-type strain. These data suggest that EnvZ^D233N^ bacteria underwent protective surface modifications that led to a reduction in binding/entry upon peptide exposure. This may be mediated via changes in the expression of LPS biosynthetic genes or genes coding for outer membrane proteins such as transporters, as detected in our transcriptomic analysis of the EnvZ^D233N^ mutant (Fig. 3). Intriguingly, however, the EnvZ^D233N^ mutant strain is not resistant to other positively charged AMPs such as polymyxin B and melittin. This indicates that surface modifications of this mutant may be more complex than a simple change of net charge and are unique to the mechanism of TAT-RasGAP_317-326_ action.

In summary, while several questions remain to be addressed to better outline the mechanism of TAT-RasGAP_317-326_ bacterial resistance, our findings provide preliminary information in this direction. This knowledge, together with that from future studies, will be indispensable in the perspective of a potential clinical use of TAT-RasGAP_317-326_ peptide or derivatives.

## MATERIALS AND METHODS

### Strains and culture conditions.

E. coli strain K-12 MG1655, BY25113 and derivatives (listed in Table S1) were grown at 37°C in LB medium (10 g/L tryptone, 5g/L yeast extract, 10g/L NaCl) or Basal Medium 2 (BM2; 62 mM potassium phosphate buffer [pH 7.0], 7 mM (NH_4_)_2_SO_4_, 10 μM FeSO_4_, 0.4% [wt/v] glucose and 0.5% tryptone) with high (2 mM) or low (20 μM) concentration of magnesium (MgSO_4_) (45). Δ*envZ* and Δ*ompR* mutants come from the KEIO collection (24), Δ*ompC* Δ*ompF* double mutant was provided by Linus Sandegren, Uppsala University, Sweden (27). All other strains were generated in this study.

### TAT-RasGAP_317-326_ peptide and other antimicrobial agents.

The retro-inverse TAT-RasGAP_317-326_ peptide (amino acid sequence DTRLNTVWMWGGRRRQRRKKRG) and its N-terminal FITC-labeled derivative were provided by SBS Genetech (Beijing, China) and stored at −20°C. Melittin was provided by Enzo Life Science (Farmingdale, NY), polymyxin B, meropenem and aztreonam by Sigma-Aldrich (Burlington, MA), and gentamicin and tetracycline by Applichem (Darmstadt, Germany).

### *In vitro* selection of resistance.

Selection of resistant strains was performed by diluting 100x an overnight culture of MG1655 E. coli in LB with 0.5x MIC (4 μM) TAT-RasGAP_317-326_ along with a no peptide control culture. The E. coli cultures were grown overnight and, the following morning, diluted again 100x in the presence of the same or an increased concentration of TAT-RasGAP_317-326_. Once growth was detected in the bacterial culture exposed to increased concentration of the peptide, the culture was propagated again and the concentration of peptide was further increased. This cycle was repeated for a total of 12 passages. The resistance selection was performed four times in parallel and four resulting clones were isolated and sequenced to identify their corresponding genetic mutations. These four resistant mutant clones correspond to EnvZ mutants A, B, C, and D discussed in this manuscript.

### MIC measurement.

MIC measurement was performed using a 96-well plate assay. Overnight cultures were diluted to OD_600_ = 0.1 and grown for 1 h at 37°C. Each culture was then further diluted 200x and 10 μL of diluted culture were added to 100 μL of LB containing sequential 10-fold dilutions of AMPs or antibiotics in a 96-well plate. The plate was then incubated at 37°C for approximately 16 h and growth was quantified by OD_590_ measurement using a FLUOstar Omega microplate reader (BMG Labtech, Ortenberg, Germany). The lowest concentrations that inhibited detectable bacterial growth were defined as the MIC for the corresponding AMP or antibiotic.

### Growth tests.

Growth tests were performed either in 96-well plates or in culture tubes. For both, an overnight culture of bacteria was diluted to OD_600_ = 0.1. For 96-well plate growth tests, 100 μL of bacterial suspension were added to each well and growth was quantified by OD_590_ measurement every 15 min under regular shaking at 37°C using a FLUOstar Omega microplate reader for a total of 12 h. For growth tests in culture tubes, 1 mL of bacterial suspension was added to the tube, incubated for 1 h at 37°C with shaking before addition of different concentrations of TAT-RasGAP_317-326_ peptide. Growth was quantified via OD_600_ measurement using a Novaspec II Visible spectrophotometer (Pharmacia LKB Biotechnology, Cambridge, England).

### Whole genome sequencing.

Genomic DNA (gDNA) was extracted from strains of interest using Wizard genomic purification kit (Promega, Madison, WI). Genomic DNA was then quantified using the Qubit system (Thermo Fisher, Waltham, MA). Libraries were then produced using a Nextera XT Kit (Illumina, San Diego, CA). Quality of the libraries was controlled by Fragment Analyzer AATI (Agilent, Santa Clara, CA) and sequencing was performed using MiSeq Reagent Kits v2 on a MiSeq system (Illumina). Obtained reads were assembled with spades v. 3.11.1 (46) and mapped on reference genomes with bwa v. 0.7.17 (47). Variant calling was done with gatk 4.0.2.0 (48). Only variants supported by a minimum of 75% of the reads were considered, and the minimum sequencing depth to call a variant was set to 10. Identified SNPs were manually checked by visualizing the mapping with JBrowse (49).

### RNA sequencing.

RNA sequencing (RNA-Seq) was performed and analyzed as recently described (18). Briefly, overnight cultures were diluted to OD_600_ = 0.1, grown for 1 h, and treated or not with 10 μM TAT-RasGAP_317-326_ for 1 h. RNA was extracted with RNeasy Plus minikit (Qiagen), and any remaining DNA was removed using DNA-free DNA Removal Kit (Invitrogen, Carlsbad, CA). Integrity of the resulting RNA samples was verified by Standard Sensitivity RNA Analysis kit (Advanced Analytical, Ankeny, IA) with the Fragment Analyser Automated CE System (Labgene Scientific, Châtel-Saint-Denis, Switzerland). Preparation of the libraries and Illumina HiSeq platform (1 × 50 bp) sequencing were performed by Fasteris (Plan-les-Ouates, Switzerland). Reads were then trimmed and mapped to genome of E. coli K-12 MG1655. Normalized expression values were calculated as reads per kilobase of transcript per million mapped reads (RPKM) with edgeR (50).

### Flow cytometry.

Flow cytometry was performed as described (18). Briefly, overnight E. coli *c*ultures were diluted to OD_600_ = 0.1 and grown for 1 h at 37°C with shaking. Bacteria were then treated with 10 μM FITC-labeled TAT-RasGAP_317-326_. After 1 h of treatment, bacteria were washed with PBS and diluted 5-fold. For each sample, 10,000 events were then collected using a CytoFLEX benchtop flow cytometer (Beckman Coulter, Brea, CA). When indicated, extracellular fluorescence was quenched using 0.2% Trypan Blue (TB) before sample acquisition. *P* values were calculated using a *t* test between the indicated conditions.

### Confocal microscopy.

Overnight E. coli cultures were diluted to OD_600_ = 0.1 and grown for 1 h. Cells were then incubated for 1 h with 10 μM FITC-labeled TAT-RasGAP_317-326_ and stained with 5 μg/mL fixable FM4-64-FX (Molecular Probes, Eugene, OR). Fixation was performed using 4% paraformaldehyde solution (EMS, Hatfield, PA). DNA was then stained with DAPI and pictures were acquired on a LSM710 confocal microscope (Zeiss, Oberkochen, Germany). Image analysis was performed using ImageJ software (51).

### Electron microscopy.

Electron microscopy was performed as described (18). Briefly, bacteria treated or not with TAT-RasGAP_317-326_ were fixed with glutaraldehyde and prepared for electron microscopy by treatments with osmium tetroxide and potassium ferrocyanide before dehydration in acetone solutions and Epon infiltration. Ultrathin sections were prepared using a Leica Ultramicrotome (Leica Mikrosysteme GmbH, Vienna, Austria) and stained with uranyl acetate. Imaging was performed with a transmission electron microscope Philips CM100 (Thermo Fisher Scientific, Waltham, MA) at an acceleration voltage of 80 kV with a TVIPS TemCam-F416 digital camera (TVIPS GmbH, Gauting, Germany). Images were analyzed with ImageJ software.

### Polymerase chain reactions.

DNA sequences of interest were amplified by PCR using either a High-Fidelity Phusion DNA polymerase (Thermo Fisher Scientific) or a GoTaq G2 Hot Start Master Mix (Promega). For 50 μL reactions with the high-fidelity DNA polymerase, reactions were prepared as follows: 1x Phusion buffer, 1 μM dNTPs, 0.5 μL Phusion polymerase, 0.2 μM primers, and DNA template. The corresponding PCR program was run in a Biometra TRIO Thermocycler (Labgene Scientific, Châtel-Saint-Denis, Switzerland) as: 95°C 30 s; 35 cycles of 95°C 10 s, 60°C 30 s, 72°C (30 s/kbp product); and finally, 72°C for 10 min. For reactions with the GoTaq DNA polymerase, reactions were prepared as follows: 1x GoTaq G2 Hot Start Master Mix, 0.2 μM each primer, and DNA template. PCR was performed using the following program: 95°C 5 min; 40 cycles of 95°C 30 s, 54°C 30 s, 72°C (1 min for every kbp of product); and finally, 72°C for 10 min. PCR products were resolved on a 1% agarose gel in 0.5x tris-borate-EDTA buffer (TBE).

### Preparation of expression vectors in pSRK plasmid.

*envZ* gene (wild-type or G697A base pair mutant corresponding to D233N amino acid mutant) was amplified from genomic DNA of the corresponding strain using High-Fidelity Phusion DNA polymerase as described above with the primers EnvZ_F_SacI and EnvZ_R_XbaI that possess additional restriction sites for SacI and XbaI enzymes (New England Biolabs, Ipswich, MA). Primer sequences are listed in Table S1. The PCR product and plasmid pSRK (52) were digested with both enzymes and digested products were gel-purified using QIAquick PCR purification system (Qiagen, Hilden, Germany). Products were then ligated and transformed in TOP10 E. coli. Colonies were screened by PCR using T7_F and T7_R primers and strains with an insert of the expected size were further verified by Sanger sequencing (Microsynth, Balgach, Switzerland) using primers T7_F and T7_R. Plasmids containing the correct insert were isolated from bacteria and stored at −20°C for later use. Plasmids were then transformed into strains of interest by heat shock transformation and plated on selective medium containing 20 μg/mL gentamicin.

### Generation of point mutations and deletions in E. coli.

Point mutations were inserted in E. coli genome using a CRMAGE protocol, based on CRISPR-Cas9 technology following an established protocol (26). The online tool provided was used to design oligonucleotides coding for a guide RNA and DNA fragment containing the desired mutation to be inserted in the genome by recombination. The strain of interest was transformed with the pMA7CR_2.0 plasmid coding for the CRISPR/Cas9 machinery and a λ/RED recombinase. The strain was then electroporated with the pMAZ-SK plasmid coding for a gRNA targeting the *envZ* gene and an oligonucleotide (Oligo_CRMAGE_EnvZ_D233N) containing a part of the mutated version of *envZ*. Colonies were then screened by PCR amplification of the *envZ* gene using GoTaq polymerase and primers EnvZ_ctrl_fwd_ext and EnvZ_ctrl_rev_ext (1 μM each) followed by Sanger sequencing (Microsynth). All primers are listed in Table S1.

Gene deletion of OmpR was performed using a Red helper plasmid pKD46 as described in Datsenko and Wanner (53). We generated PCR fragments with a selectable antibiotic resistance gene and short homology extensions that allow for replacement of the chromosomal gene sequence via Red recombination. To make the PCR constructs, we used several pairs of 50- to 75-nt-long primers that included 30- to 35-nt homology extensions and 20-nt priming sequences based on the plasmids pKD3 as template (Table S1). Plasmid isolation was performed with a PureYield Plasmid Miniprep kit (Promega). The 1.1 kbp PCR product was purified with the Wizard Plus SV Minipreps DNA Purification System (Promega), treated with DpnI (New England Biolabs) for 1 h at 37°C and then transformed into electrocompetent MG1655 (EnvZ^D233N^) carrying pKD46. To make electrocompetent cells, the strain of interest was grown in 5 mL LB culture with 100 μg/mL ampicillin and 1 mM L-arabinose at 30°C to OD_600_ ~ 0.6, concentrated 100-fold, and then washed three times with ice-cold sterile H_2_O. For electroporation, an aliquot of 100 μL of the cell suspension was mixed with 50 ng of purified PCR product. The mixture was placed in a pre-chilled sterile electroporation cuvette (1 mm electrode gap, Bio-Rad, Hercules, CA) and immediately pulsed by use of a Bio-Rad Gene Pulser (1.8 kV, 200 W, 25 μF). The electroporated bacterial suspension was incubated at room temperature for 18 h in the presence of 1 mL LB medium. Cells were then spread on LB agar containing 25 μg/mL chloramphenicol. Colony PCR was used to identify mutant clones using forward and reverse primers (listed in Table S1) to verify simultaneous loss of the parental chromosomal sequence and gain of the new mutant-specific fragment.
