# Supplementary material for: The EnvZ/OmpR Two-Component System Regulates the Antimicrobial Activity of TAT-RasGAP317-326 and the Collateral Sensitivity to Other Antibacterial Agents
Source: Microbiol Spectr. 2022 May 17;10(3):e02009-21. doi: 10.1128/spectrum.02009-21 (PMC9241736; doi:10.1128/spectrum.02009-21)
Supplement: SUPPLEMENTAL FILE 2 — Supplemental material. Download spectrum.02009-21-s0002.pdf, PDF file, 0.3 MB [file spectrum.02009-21-s0002.pdf]

Table S1: Strains, plasmids and oligonucleotides used in this study.

Strains

Name	Genotype	Origin
MG1655 (ATCC47076)		ATCC
Mutant A	MG1655 *envZ* ^{G697A}	This study
Mutant B	MG1655 *envZ* ^{T722G} *basS* ^{A371C}	This study
Mutant C	MG1655 *envZ* ^{D273A} *rfaY* ^{649delA}	This study
Mutant D	MG1655 *envZ* ^{P248A} *yrbL* ^{G445C}	This study
BY25113	*lacI* q *rrnB* T14 Δ*lacZ* WJ16 *hsdR514* Δ*araBAD* AH33 Δ*rhaBAD* LD78	Baba et al., 2006
ΔEnvZ	BY25113 *envZ::kan*	Baba et al., 2006
ΔOmpC ΔOmpF	MG1655 *ompC* ::FRT scar *ompF* ::FRT scar	Adler et al., 2016
ΔOmpR	BY25113 *ompR::kan*	Baba et al., 2006
EnvZ ^{D233N} ΔOmpC ΔOmpF	MG1655 *envZ* ^{G697A} *ompC* ::FRT scar *ompF* ::FRT scar	This study
EnvZ ^{D233N} ΔOmpR	MG1655 *envZ* ^{G697A} *ompR::Kan*	This study

Plasmids

Name	Details	Resistance	Origin
pSRK	Expression vector, IPTG inducible, empty	Gentamicin	Khan et al., 2008
pSRK-EnvZ	Expression vector, IPTG inducible, for *envZ* ^{WT} expression	Gentamicin	This study
pSRK-EnvZ ^{D233N}	Expression vector, IPTG inducible, for *envZ* ^{G697A} expression	Gentamicin	This study
pKD46	Plasmid allowing expression of a λ/RED recombinase	Ampicillin	Datsenko and Wanner, 2000
pKD3	Plasmid used as template for knock-out cassette amplification	Chloramphenicol	Datsenko and Wanner, 2000
pMA7CR_2.0	Plasmid coding for CRISPR-Cas9 and a λ/RED recombinase	Ampicillin	Ronda et al., 2016
pMAZ-SK	Plasmid coding for gRNAs	Kanamycin	Ronda et al., 2016
pMAZ-SK-gRNA-EnvZ	Plasmid coding for gRNA targeting *envZ* gene	Kanamycin	This study

Oligonucleotides

Name	Sequence	Purpose	Origin
Oligo_gRNA_EnvZ	GTGATAGAGATACTGAGCACCCGTGCCTTTAACCATATGGGTTTTAGAGCT AGAAATAGC	CRMAGE mutagenesis of EnvZ	This study
Oligo_CRMAGE_EnvZ_D233N	ACCATATGGCTGCTGGTGTTAAGCAACTGGCGGATAACCGCACGCTGCTG ATGGCGGGGGTAAGTCACGA	CRMAGE mutagenesis of EnvZ	This study
P23_pMAZ-SK	GTTTTAGAGCTAGAAAT	Cloning to insert EnvZ specific gRNA in pMAZ-SK	Ronda et al., 2016
P24_pMAZ-SK	GTGCTCAGTATCTCT	Cloning to insert EnvZ specific gRNA in pMAZ-SK	Ronda et al., 2016
EnvZ_F_SacI	AAAAAAGAGCTCATGAGGCGATTGCG	Cloning of EnvZ in pSRK plasmid	This study
EnvZ_R_XbaI	AAAAAATCTAGATTACCCTTCTTTTGTCGTGC	Cloning of EnvZ in pSRK plasmid	This study

EnvZ_ctrl_fwd_ext	GCTACGTCTTTGTACCGG	Amplification of EnvZ for control/sequencing	This study
envZ_ctrl_rev_ext	CCAGTATCTTATAGAAAGCAA	Amplification of EnvZ for control/sequencing	This study
OmpR_ko_fwd	TTTAAGAATACACGCTTACAAATTGTTGCGAACCTTTGGGAGTACAAACAGT GTAGGCTGGAGCTGCTTC	Knock-out of OmpR	This study
OmpR_ko_rev	GAGCAATAACGTACGGGCAAATGAACTTCGTGGCGAGAAGCGCAATCGCC CATATGAATATCCTCCTTAG	Knock-out of OmpR	This study
OmpR_ctrl_fwd	TTTAAGAATACACGCTTACA	Control of OmpR knock-out	This study
OmpR_ctrl_rev	GAGCAATAACGTACGG	Control of OmpR knock-out	This study
T7_F	TAATACGACTCACTATAGGG	Control of insert size and sequence in pSRK plasmid	Khan et al., 2008
T7_R	TAGTTATTGCTCAGCGGTGG	Control of insert size and sequence in pSRK plasmid	Khan et al., 2008

Table S2. Genes with higher expression in $EnvZ^{D233N}$ mutant compared to wild-type in absence of peptide. Reads Per Kilobase of transcript per Million mapped reads (RPKM) are shown for wild-type (WT_untreated) and $EnvZ^{D233N}$ ($EnvZ^{D233N}$_untreated). The Log in base 2 of the fold-change between expression in $EnvZ^{D233N}$ and wild-type ($Log_2FC\_EnvZ^{D233N}\_vs\_WT$) was calculated and genes showing a value higher than 2 (corresponding to at least a 4-fold increase in expression) are shown in this table.

WT_untreated	$EnvZ^{D233N}$_untreated	Gene	Product	Locus_tag	$Log_2FC\_EnvZ^{D233N}\_vs\_WT$
335.486	3019.932	tatE	twin arginine protein translocation system - TatE protein	b0627	3.1702
17.829	74.625	rlmH	23S rRNA m(3)psi1915 methyltransferase	b0636	2.0654
145.640	1106.019	ybhB	putative kinase inhibitor	b0773	2.9249
15.613	68.872	narZ	nitrate reductase Z subunit alpha	b1468	2.1412
114.016	462.693	gadC	L-glutamate:4-aminobutyrate antiporter	b1492	2.0208
12.882	85.311	ydeI	BOF family protein YdeI	b1536	2.7274
2.298	31.203	rspB	putative zinc-binding dehydrogenase RspB	b1580	3.7633
21.451	249.601	rspA	mandelate racemase/muconate lactonizing enzyme family protein RspA	b1581	3.5405
43.994	1206.506	asr	acid shock protein	b1597	4.7774
105.960	695.482	hdhA	7-alpha-hydroxysteroid dehydrogenase	b1619	2.7145
207.522	1064.339	anmK	anhydro-N-acetylmuramic acid kinase	b1640	2.3586
34.811	174.782	ydhI	DUF1656 domain-containing protein YdhI	b1643	2.3279
49.018	221.890	sufB	Fe-S cluster scaffold complex subunit SufB	b1683	2.1785
32.775	132.834	sufA	iron-sulfur cluster insertion protein SufA	b1684	2.0189
45.967	343.861	yecD	putative hydrolase	b1867	2.9032
25.208	141.163	yfaZ	putative porin YfaZ	b2250	2.4854
17.270	115.882	valX	tRNA-Val	b2402	2.7463
0	63.935	valY	tRNA-Val	b2403	N/A
29.705	165.887	galP	galactose:H(+) symporter	b2943	2.4814
25.924	629.007	yhcN	DUF1471 domain-containing stress-induced protein YhcN	b3238	4.6007
8.446	308.068	yhdV	lipoprotein YhdV	b3267	5.1888
20.517	109.174	rhsB	rhs element protein RhsB	b3482	2.4118
262.932	2080.555	yiaD	PF13488 family lipoprotein YiaD	b3552	2.9842
3.093	102.917	yiaY	L-threonine dehydrogenase	b3589	5.0566
13.551	310.021	waaJ	UDP-glucose:(glucosyl)LPS alpha-1,2-glucosyltransferase	b3626	4.5159
13.056	62.215	metE	cobalamin-independent homocysteine transmethylase	b3829	2.2526
6.799	60.900	yjeM	putative transporter YjeM	b4156	3.1630
0	122.872	ymdF	conserved protein YmdF	b4518	N/A
0	125.815	yddY	protein YddY	b4746	N/A

Table S3. Genes with lower expression in EnvZ^{D233N} mutant compared to wild-type in absence of peptide. Reads Per Kilobase of transcript per Million mapped reads (RPKM) are shown for wild-type (WT_untreated) and EnvZ^{D233N} (EnvZ^{D233N}_untreated). The Log in base 2 of the fold-change between expression in EnvZ^{D233N} and wild-type (Log$_2$FC_EnvZ^{D233N}_vs_WT) was calculated and genes showing a value lower than -2 (corresponding to at least a 4-fold decrease in expression) are shown in this table.

WT_untreated	EnvZ^{D233N}_untreated	Gene	Product	Locus_tag	Log$_2$FC_EnvZ^{D233N}_vs_WT
99.100	24.252	fhuA	ferrichrome outer membrane transporter/phage receptor	b0150	-2.031
40.664	8.537	rrrD	DLP12 prophage; lysozyme	b0555	-2.252
16792.483	286.750	borD	DLP12 prophage; prophage lipoprotein BorD	b0557	-5.872
2146.866	32.788	ompT	outer membrane protease VII (outer membrane protein 3b)	b0565	-6.033
1017.518	81.330	cstA	carbon starvation protein A	b0598	-3.645
78.094	2.720	chiP	chitobiose outer membrane channel	b0681	-4.844
28.372	5.594	lysZ	tRNA-Lys	b0748	-2.342
3307.450	715.254	bssR	regulator of biofilm formation	b0836	-2.209
5092.061	33.074	ompF	outer membrane porin F	b0929	-7.266
169.922	39.083	putP	proline:Na(+) symporter	b1015	-2.120
77.115	8.157	flgB	flagellar basal-body rod protein FlgB	b1073	-3.241
47.455	10.048	flgC	flagellar basal-body rod protein FlgC	b1074	-2.240
459.452	93.993	ymfE	e14 prophage; uncharacterized protein YmfE	b1138	-2.289
32.698	7.723	chaA	Na(+)/K(+):H(+) antiporter ChaA	b1216	-2.082
27.605	5.483	tonB	Ton complex subunit TonB	b1252	-2.332
51.378	9.208	yncE	PQQ-like domain-containing protein YncE	b1452	-2.480
148.508	5.757	pqqL	putative zinc peptidase	b1494	-4.689
51.084	1.894	yddB	putative TonB-dependent receptor	b1495	-4.753
39.981	9.078	yddA	ABC transporter family protein YddA	b1496	-2.139
29.606	2.398	asnU	tRNA-Asn	b1986	-3.626
816.381	101.458	pmrD	signal transduction protein PmrD	b2259	-3.008
287.363	19.470	hisJ	histidine ABC transporter periplasmic binding protein	b2309	-3.884
56.737	13.366	plsY	putative glycerol-3-phosphate acyltransferase	b3059	-2.086
73.797	8.586	sstT	serine/threonine:Na(+) symporter	b3089	-3.103
27.854	6.492	ygjV	inner membrane protein	b3090	-2.101
457.448	62.021	yqjA	DedA family protein YqjA	b3095	-2.883
700.212	133.814	mzrA	modulator protein MzrA	b3096	-2.388
66.147	15.185	yhdU	DUF2556 domain-containing protein YhdU	b3263	-2.123
1179.975	228.048	malT	DNA-binding transcriptional activator MalT	b3418	-2.371

169.348	16.174	waaU	putative ADP-heptose:LPS heptosyltransferase 4	b3623	-3.388
48.197	6.060	waaZ	lipopolysaccharide core biosynthesis protein WaaZ	b3624	-2.992
2321.077	559.526	ivbL	ilvBN operon leader peptide	b3672	-2.053
36.125	8.358	spf	small regulatory RNA Spot 42	b3864	-2.112
124.308	29.694	pyrL	pyrBI operon leader peptide	b4246	-2.066
32.846	7.843	fecC	ferric citrate ABC transporter membrane subunit FecC	b4289	-2.066
62.709	7.601	fecB	ferric citrate ABC transporter periplasmic binding protein	b4290	-3.044
53.308	3.161	fecA	ferric citrate outer membrane transporter	b4291	-4.076
48.765	10.679	yjhQ	KpLE2 phage-like element; putative acetyltransferase TopAI antitoxin	b4307	-2.191
52.768	7.081	fimA	type 1 fimbriae major subunit	b4314	-2.898
39.063	8.211	fimI	putative fimbrial protein FimI	b4315	-2.250
49.846	7.028	fimC	type 1 fimbriae periplasmic chaperone	b4316	-2.826
148.255	35.190	fimG	type 1 fimbriae minor subunit FimG	b4319	-2.075
26.973	5.543	fhuF	hydroxamate siderophore iron reductase	b4367	-2.283
422.424	50.266	cyaR	small regulatory RNA CyaR	b4438	-3.071
2301.664	427.314	ybdD	conserved protein YbdD	b4512	-2.429
46.008	6.186	yniD	uncharacterized protein YniD	b4535	-2.895
31.755	7.511	ymgJ	uncharacterized protein YmgJ	b4594	-2.080
151.446	33.555	ryfD	small regulatory RNA RyfD	b4609	-2.174
52.616	10.536	mgrR	small regulatory RNA MgrR	b4698	-2.320
1149.332	257.574	ybgU	protein YbgU	b4735	-2.158
39.714	7.592	yqhI	protein YqhI	b4755	-2.387
68.289	13.497	ysdD	protein YsdD	b4757	-2.339

Table S4: Genes with increased expression upon 10 µM TAT-RasGAP$_{317-326}$ treatment in EnvZ^{D233N} mutant but not in wild-type. Reads Per Kilobase of transcript per Million mapped reads (RPKM) are shown for wild-type treated with TAT-RasGAP$_{317-326}$ (WT_TAT-RasGAP) or not (WT_untreated) and EnvZ^{D233N} treated or not. The Log in base 2 of the fold-change between expression in EnvZ^{D233N} treated with the peptide and in absence of the treatment (Log$_2$FC_EnvZ^{D233N}_TAT-RasGAP_vs_untreated) was calculated and genes showing a value higher than 2 (corresponding to at least a 4-fold increase in expression) are shown in this table. Genes for which the Log$_2$FC_WT_TAT-RasGAP_vs_untreated was higher than 2 were excluded from this list.

WT_TAT-RasGAP	WT_untreated	EnvZ^{D233N}_TAT-RasGAP	EnvZ^{D233N}_untreated	Gene	Product	Locus_tag	Log$_2$FC_EnvZ^{D233N}_TAT-RasGAP_vs_untreated
6.317	19.339	109.592	15.130	leuC	3-isopropylmalate dehydratase subunit LeuC	b0072	2.857
19.312	9.530	37.289	3.170	leuB	3-isopropylmalate dehydrogenase	b0073	3.556
25.513	21.172	95.806	12.441	leuA	2-isopropylmalate synthase	b0074	2.945
432.584	194.677	628.831	154.856	hemL	glutamate-1-semialdehyde aminotransferase	b0154	2.022
111.665	42.141	190.589	24.121	fadE	acyl-CoA dehydrogenase	b0221	2.982
96.269	28.196	161.924	37.143	fadM	thioesterase III	b0443	2.124
128.930	40.664	36.195	8.537	rrrD	DLP12 prophage; lysozyme	b0555	2.084
692.965	2146.866	783.765	32.788	ompT	outer membrane protease VII (outer membrane protein 3b)	b0565	4.579
1359.119	369.503	2318.247	327.888	ybhF	putative ABC exporter ATP binding subunit	b0794	2.822
98.386	77.048	571.888	100.865	ybhG	HlyD_D23 family protein YbhG	b0795	2.503
100.870	57.619	275.904	30.459	cecR	DNA-binding transcriptional dual regulator CecR	b0796	3.179
7.532	17.188	32.575	6.884	lysO	L-lysine exporter	b0874	2.243
47.411	12.141	11.197	1.457	csgF	curli assembly component	b1038	2.942
7.418	7.387	41.624	8.098	ymgD	PF16456 family protein YmgD	b1171	2.362
69.256	51.187	99.224	20.595	ortT	orphan toxin OrtT	b1445	2.268
246.609	258.860	1273.830	265.813	rstA	DNA-binding transcriptional regulator RstA	b1608	2.261
130.965	113.477	379.911	82.538	ydhC	putative transporter YdhC	b1660	2.203
1824.067	779.966	2658.053	424.323	mgrB	PhoQ kinase inhibitor	b1826	2.647
307.542	267.610	1862.402	278.180	hisG	ATP phosphoribosyltransferase	b2019	2.743
42.564	39.512	217.876	39.189	hisD	histidinal/histidinol dehydrogenase	b2020	2.475
28.657	57.249	281.244	59.547	hisC	histidinol-phosphate aminotransferase	b2021	2.240
123.681	32.958	109.041	18.204	lpxT	Kdo2-lipid A phosphotransferase	b2174	2.583
28.941	8.160	22.923	4.724	yfcP	putative fimbrial protein YfcP	b2333	2.279
46.699	17.858	35.441	7.231	tadA	tRNA adenosine(34) deaminase	b2559	2.293
151.320	52.655	72.390	17.750	ygdR	DUF903 domain-containing lipoprotein YgdR	b2833	2.028
41.459	14.622	18.928	2.415	glpE	thiosulfate sulfurtransferase GlpE	b3425	2.971

93.410	48.097	552.041	23.489	livG	branched chain amino acid/phenylalanine ABC transporter ATP binding subunit	b3455	4.555
39.470	40.916	190.302	18.785	ilvN	acetohydroxy acid synthase I subunit IlvN	b3670	3.341
90.411	63.612	345.232	32.113	ilvB	acetohydroxy acid synthase I subunit IlvB	b3671	3.426
32.419	23.373	47.263	9.454	phoU	negative regulator of the pho regulon	b3724	2.322
39.306	11.785	38.449	8.521	pstA	phosphate ABC transporter membrane subunit PstA	b3726	2.174
661.157	234.380	1546.709	162.888	ilvM	acetolactate synthase II subunit IlvM	b3769	3.247
54.352	18.686	64.320	11.219	fadA	3-ketoacyl-CoA thiolase	b3845	2.519
74.800	21.276	85.206	7.655	fadB	dodecenoyl-CoA delta-isomerase, enoyl-CoA hydratase, 3-hydroxybutyryl-CoA epimerase, 3-hydroxyacyl-CoA dehydrogenase	b3846	3.477
43.186	36.125	33.870	8.358	spf	small regulatory RNA Spot 42	b3864	2.019
38.059	17.620	26.376	5.815	yjcB	uncharacterized protein YjcB	b4060	2.181
203.441	422.424	227.153	50.266	cyaR	small regulatory RNA CyaR	b4438	2.176
929.551	250.006	1636.565	216.772	ymgI	uncharacterized protein YmgI	b4593	2.916
299.050	402.583	5400.472	117.903	ilvX	uncharacterized protein IlvX	b4669	5.517
442.832	574.666	14760.427	299.191	mgtL	leader peptide MgtL	b4702	5.625

Table S5: Genes with decreased expression upon 10 µM TAT-RasGAP$_{317-326}$ treatment in EnvZ^{D233N} mutant but not in wild-type. Reads Per Kilobase of transcript per Million mapped reads (RPKM) are shown for wild-type treated with TAT-RasGAP$_{317-326}$ (WT_TAT-RasGAP) or not (WT_untreated) and EnvZ^{D233N} treated or not. The Log in base 2 of the fold-change between expression in EnvZ^{D233N} treated with the peptide and in absence of the treatment (Log$_2$FC_EnvZ^{D233N}_TAT-RasGAP_vs_untreated) was calculated and genes showing a value lower than -2 (corresponding to at least a 4-fold decrease in expression) are shown in this table. Genes for which the Log$_2$FC_WT_TAT-RasGAP_vs_untreated was lower than -2 or higher than 2 were excluded from this list.

WT_TAT-RasGAP	WT_untreated	EnvZ^{D233N}_TAT-RasGAP	EnvZ^{D233N}_untreated	Gene	Product	Locus_tag	Log$_2$FC_EnvZ^{D233N}_TAT-RasGAP_vs_untreated
21.643	35.202	11.025	48.870	ygcO	putative 4Fe-4S cluster-containing protein	b2767	-2.148182384
16.491	57.150	16.441	141.563	tdcE	2-ketobutyrate formate-lyase/pyruvate formate-lyase 4	b3114	-3.106041642
21.359	62.992	11.844	93.492	hdeD	acid-resistance membrane protein	b3511	-2.980707882
0	0	28.439	125.815	yddY	protein YddY	b4746	-2.145369198
